# Decision-making competence in patients with depression and a history of suicide attempt: A systematic review

**DOI:** 10.1192/j.eurpsy.2024.301

**Published:** 2024-08-27

**Authors:** I. Pavlou, V. Efstathiou, A. Karvouni, I. Theodoridou, N. Smyrnis, I. Michopoulos, R. Gournellis, A. Papadopoulou

**Affiliations:** ^1^MSc “Liaison Psychiatry: Integrative Care of Physical and Mental Health”; ^2^Psychology Department, National and Kapodistrian University of Athens; ^3^Second Department of Psychiatry, National and Kapodistrian University of Athens, “Attikon” University General Hospital, Athens, Greece

## Abstract

**Introduction:**

Decision-making is a complex process, and little is known about the various elements that comprise it. Recent literature on neurocognitive deficits in patients with a history of suicidality has highlighted that impaired (non-adaptive) decision-making is one of the most consistent deficits in individuals with a history of suicidality.

**Objectives:**

This study aims to systematically review the available evidence on decision- making capacity in depressed patients with a history of suicide attempts.

**Methods:**

A systematic search was conducted in PubMed, Psycnet, Elsevier and Scopus with additional searching through bibliographic references. This search was performed until the 31st of August 2022 and provided information on decision-making capacity in relation to suicidality and depression.

**Results:**

The literature review provided 377 references, the titles and abstracts of which were reviewed for relevance to this study and the entry criteria set. The review of the title and abstract of these studies resulted in 50 articles that were potentially relevant to the study topic and a further review was then conducted to re-examine the selected studies and articles, which resulted in the final selection of 20 studies. The outcome measure used by the majority of studies as a measure of decision-making ability was the IOWA Gambling Task (IGT), in which the performance of patients with a history of depression and self-harm in most studies was significantly worse than that of healthy controls. Some methodological characteristics of the studies included in this review complicated the interpretation of the results, such as the sample size and characteristics of each study.

**Conclusions:**

Decision-making ability shows alterations in patients with a history of suicidality and depression, confirming the findings of previous studies. Furthermore, an impaired or dysfunctional decision-making ability may potentially be a predictor of suicidal behaviour in patients with depression, a possibility that could be a reason for further research in this field, both in the context of investigating predictors and in developing appropriate treatments for these patients.

**Disclosure of Interest:**

None Declared

